# Viral and Host Characteristics of Recent and Established HIV-1 Infections in Kisumu based on a Multiassay Approach

**DOI:** 10.1038/srep37964

**Published:** 2016-11-29

**Authors:** Newton Otecko, Seth Inzaule, Collins Odhiambo, George Otieno, Valarie Opollo, Alex Morwabe, Kennedy Were, Kenneth Ndiege, Fredrick Otieno, Andrea A. Kim, Clement Zeh

**Affiliations:** 1 Kenya Medical Research Institute/U.S. CDC Research and Public Health Collaboration (KEMRI/CDC) and KEMRI Center for Global Health Research, Kisumu, Kenya; 2 U.S. Centers for Disease Control and Prevention (CDC-Kenya, Division of HIV/AIDS Prevention) and the KEMRI/CDC Research and Public Health Collaboration, Kisumu, Kenya

## Abstract

Integrated approaches provide better understanding of HIV/AIDS epidemics. We optimised a multiassay algorithm (MAA) and assessed HIV incidence, correlates of recent infections, viral diversity, plus transmission clusters among participants screened for Kisumu Incidence Cohort Study (KICoS1) (2007–2009). We performed BED-CEIA, Limiting antigen (LAg) avidity, Biorad avidity, and viral load (VL) tests on HIV-positive samples. Genotypic analyses focused on HIV-1 *pol* gene. Correlates of testing recent by MAA were assessed using logistic regression model. Overall, 133 (12%, 95% CI: 10.2–14.1) participants were HIV-positive, of whom 11 tested recent by MAA (BED-CEIA OD-n < 0.8 + LAg avidity OD-n < 1.5 + VL > 1000 copies/mL), giving an incidence of 1.46% (95% CI: 0.58–2.35) per year. This MAA-based incidence was similar to longitudinal KICoS1 incidence. Correlates of testing recent included sexually transmitted infection (STI) treatment history (OR = 3.94, 95% CI: 1.03–15.07) and syphilis seropositivity (OR = 10.15, 95% CI: 1.51–68.22). Overall, HIV-1 subtype A (63%), D (15%), C (3%), G (1%) and recombinants (18%), two monophyletic dyads and intrinsic viral mutations (V81I, V81I/V, V108I/V and K101Q) were observed. Viral diversity mirrored known patterns in this region, while resistance mutations reflected likely non-exposure to antiretroviral drugs. Management of STIs may help address ongoing HIV transmission in this region.

Sub-Saharan Africa bears the greatest burden of the HIV/AIDS epidemic. In 2014, the number of people living with HIV in this region was approximately 25.8 million, and about 1.4 million were newly infected, accounting for two thirds of new daily infections worldwide^1^. In Kenya, the overall prevalence was 5.6% by 2012, with Nyanza region of western Kenya recording the highest prevalence (15.1%)^2^.

Recently, Kenyan leaders launched a visionary program employing geographic and populations targeted measures to achieve zero new HIV infections by 2030^3^. While prevalence is a basic epidemiological tool in countries with >1% HIV prevalence, it is a poor measurement of finer changes in an epidemic^4^, and would not sufficiently support policies like the Kenyan ‘prevention-revolution’.

Characterising new HIV infections in a population provides better insights about the epidemic, including the impact of the disease on the public’s health and related prevention efforts. It also allows for identification of factors responsible for onward transmission of the disease^5^,^6^, an important prerequisite for implementing robust prevention programs. Owing to the cost-prohibitive nature of longitudinal HIV incidence studies^7^, several serological assays have been developed for cross-sectional estimation of HIV incidence. However, these assays falsely classify some established infections as recent, thereby overestimating population incidence^8^,^9^,^10^. A multi-assay algorithm (MAAs), is a hierarchical recent infection testing algorithm which employs one or more serological incidence assays, combined with viral load and other available non-serological biomarkers such as metabolites for antiretroviral drug (ARV) drugs, to reduce misclassification of chronic HIV infections as recent on the serological incidence assays^11^,^12^,^13^,^14^,^15^,^16^,^17^,^18^,^19^. Serological incidence assays target a biomarker that is indicative of early phase HIV infection^5^,^20^. However, changes in such biomarkers over time often vary per person^21^, hence the use of incidence assays at population rather than individual level.

Apart from incidence testing, techniques like genetic sequencing provide insights on vital viral properties that could boost understanding of HIV epidemics. HIV-1 exhibits high diversity with nine distinct subtypes: A–D, F–H, J and K and several circulating recombinant forms (CRFs) which show distinct geographical distribution^22^. In Kenya, subtype A is most predominant, however, subtypes C, D, G and CRFs are also common^23^,^24^,^25^. Viral diversity remains a challenge to HIV diagnosis, treatment, and other biomedical interventions^26^. Hence knowledge and continuous monitoring of circulating viral strains is an indispensible requisite for effective HIV prevention.

Current technological advances have made multi-pronged assessment integrating serological and molecular viral data, together with host’s clinical, demographic and socio-behavioral indices, easily attainable. This could provide highly useful and timely information on which to base formulation and review of transmission prevention initiatives. In this paper, we aimed to optimise an MAA strategy and utilise viral and host data to characterise MAA-identified recent and established HIV infections among participants screened for Kisumu HIV Incidence Cohort Study (KICoS)^27^.

## Results

### General characteristics of study participants

Of the 1106 participants, the median age was 21 years (range 16–34 years), and HIV-1 infection prevalence was 12% (n = 133) (95% CI: 10.2–14.1). Among the 133 HIV positives, 98 (73.7%) were females and 35 (26.3%) males, with an overall median age of 23 years (range 16–34 years) (Table 1). Briefly, fewer participants (286/1053, 27.2%) had acquired post secondary education and a majority (833/1099, 75.8%) had never been married. While most participants reported having had no previous treatment for sexually transmitted infections (STIs), a majority (66.2%) of the HIV infected individuals had HSV-2 co-infection. We noticed a reduced rate of reporting, especially pertaining to sexual behaviour questions, but a majority (168/235, 71.5%) of those who responded reported having received money in exchange for sex, while condom use during sex in the previous three months was low, especially among HIV positive individuals (43/110, 39.1) (Table 1).

### Recent and established HIV-1 infections

Of the HIV-positive samples (n = 133), a total of 8 samples were excluded from the MAAs evaluation due to missing Biorad and BED-CEIA results (5 of which also lacked LAg avidity results) occasioned by sample depletion in preceding tests, resulting into 125 samples with complete incidence assays and VL results.

Considering single assays, Biorad avidity had the highest proportion of recent infections compared to the other two assays i.e. 20.0% versus 15.2% and 13.2% for BED-CEIA and LAg avidity respectively. Similarly, there was a trend where MAAs with single incidence assay plus VL generally had higher estimates of recent infections compared to MAAs with more than one incidence assay (Table 2). Narrowing our focus on the MAAs in the last category reported above, we reviewed the incidence estimates against the KICoS1 incidence (1.4%) estimated prospectively and selected the MAA in which recent infections were defined by BED-CEIA OD-n < 0.8 + LAg OD-n < 1.5 + VL > 1000 copies/mL, giving 11 (8.8%, 95% CI: 5.0–15.1) recent infections and incidence of 1.46% (95% CI: 0.58–2.35) per year.

Of the 125 samples used in evaluating the MAAs, 114 (91.2%) (12 recent and 102 established) were concordantly classified by BED-CEIA and LAg avidity assays (Kappa score of 0.635 (P = 0.001, 95% CI: 0.429–0.841) and Pearson’s phi coefficient (ϕ) of 0.638 (P = 0.001)) (Supplementary Table S1). One of these 12 recent infections, with VL of 761 copies/mL, was reclassified as established based on the MAA’s criteria. We show the general overview of the classification of all the 125 samples by all the five parameters and by the selected MAA (Supplementary Fig. S1).

We further analysed the performance of the three incidence assays on 144 samples that were classified as established by the MAA. Biorad avidity had the highest rate of misclassification of samples as recent, followed by BED-CEIA (Table 3). In addition, VL mean values were lower among samples classified as recent infections than the established infections, for all the three incidence assays. However the differences were not statistically significant (Table 3). Although A, D and AD were the subtypes misclassified as recent, statistical analysis showed that overall misclassification by the three incidence assays were not significantly linked with viral diversity, Chi-square p-values > 0.05 (Supplementary Fig. S2).

### Characteristics of individuals with multiassay algorithm-determined recent infections

Among individuals tested as recently infected on the BED + Lag + VL MAA, 7/11 (63.6%) were females, a similar number were <25 years old, and a majority (8/11, 72.7%) had never married. The most prevalent HIV subtype was A (6/11, 54.5%), followed by C and AD (both with 2/11, 18.2%), and lastly D (1/11, 9.1%) (Supplementary Table S2). In the regression model for HIV recent infection versus HIV negatives, after controlling for age, gender, sex for gifts, history of STI treatment, syphilis and HSV-2, only history of STI treatment and syphilis sero-positivity explained the recent infections in this population (Table 4). Persons reporting past treatment for STI were nearly 4 times more likely to be recently infected than those never treated for STI (OR = 3.94; 95% CI: 1.03–15.07), while those who tested positive for syphilis were ten times more likely to be recently infected compared to the syphilis negative (OR = 10.15; 95% CI: 1.51–68.22). Only history of STI treatment was linked to recent infection in the second model comparing recent against established infections (OR = 9.91; 95% CI: 1.55–63.46) (Supplementary Table S3).

### Viral subtyping analysis

Of the 133 HIV-positive samples, a total of 100 (75.2%) samples were successfully genotyped (GenBank accession numbers KX306376-KX306475). The remaining 33 samples included: 14 with insufficient plasma quantities, 5 with viral load <1000 copies/mL (the amplification sensitivity threshold for sequencing), 9 with undetectable viral load, and 5 that failed amplification.

**Figure 1:**
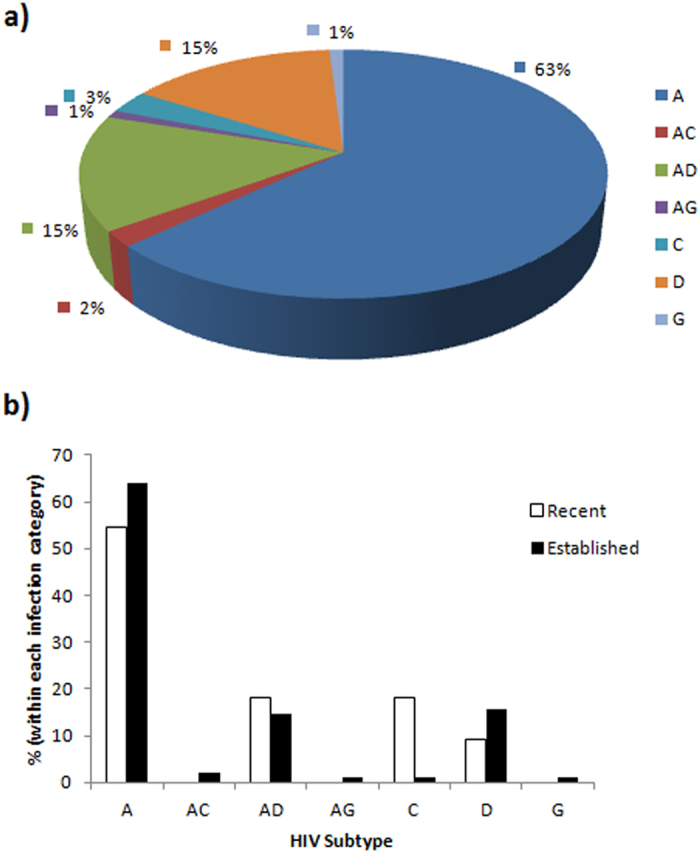
HIV subtype distribution among study participants (n = 100). (**A**) overall distribution. (**B**) Categorised by recent (open bars, n = 11) and established (closed bars, n = 89) HIV infections, Kisumu Incidence Cohort Study (KICoS): 2007–2009.

Overall, the predominant HIV-1 subtype was A (63%), followed by D (15%), C (3%) and G (1%). Recombinant variants constituted 18% of all the samples with AD dominating at 15%, followed by AC (2%) and AG (1%) (Fig. 1a). Subtypes G, AC and AG, were only present in established infections (Fig. 1b). There was no significant difference in subtype distribution between recent and established infections.

### HIV transmission network analysis

**Figure 2:**
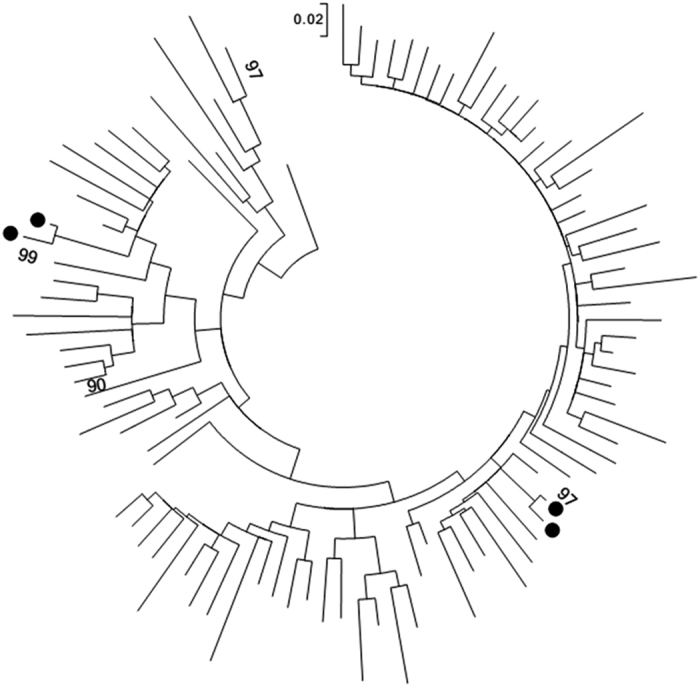
Molecular phylogenetic analysis for HIV transmission clustering. Evolutionary history was inferred using Maximum Likelihood method based on the Tamura-Nei model in Mega v.7.0. The tree with the highest log likelihood is shown. Nodes with high bootstrap values indicated. Black dots represent individuals in transmission clusters.

Evaluation of evolutionary relatedness among 98 samples (after removing two sequences with sequence quality issues) revealed two monophyletic dyads (Fig. 2) of HIV-1 subtype A and AD. Each dyad had an older male and a younger female. One dyad had single individuals while individuals in the other dyad were both in married relationships. All the individuals had MAA-classified established infections characterised by higher VL in males compared to females. Discordant variable outcomes within dyads, for instance condom use, imply the possibility of transmission networks being larger than captured by this study. Additionally, the small size of the dyads could be due to limited sample size and not an indication of transmission pairs (Supplementary Table S4).

### Viral resistance mutations analysis

Observed viral mutations included V81I and V81I/V in the protease gene, and V108I/V and K101Q in the reverse transcriptase gene, all of which were intrinsic mutations not associated with ARV exposure. Four of the five individuals bearing the mutations were females, while two individuals (one male) had recent infections (Table 5).

## Discussion

This is one of the few applications of a multifactorial strategy and the recent approaches and recommendations for cross-sectional HIV incidence testing algorithms in Africa^16^,^17^. We optimised an MAA that identified potential recent HIV infections in the study population. We then analysed host factors and viral molecular properties characterising this population.

From our analysis, single incidence assays or MAAs with single incidence assay plus one or two non-serological biomarkers seemed to give poor outcomes compared to MAAs integrating at least two incidence assays and one or more non-serological biomarkers. This pattern generally corroborates the findings from previous evaluations of single incidence assays and MAAs in different setups^8^,^12^,^17^,^28^,^29^. Single incidence assays in our study gave more than twofold higher incidence estimates compared to the longitudinal KICoS1 incidence. This emphasises the inappropriateness of using the current incidence assays singly in cross-sectional incidence studies^9^,^13^. The selected MAA comprised of BED-CEIA, a relatively inexpressive and widely used assay^28^, plus LAg avidity, an assay with commendable performance in different epidemics, and VL, a test that is currently recommended for incidence MAAs^9^. The concordance between BED-CEIA and LAg avidity assays was statistically significant, with modest Pearson and Kappa statistical values. With expansion of capacity for dry blood spot specimens and point of care VL testing, such MAA will even be more attainable in resource limited settings.

In our study, HIV prevalence was approximately 12% (95% CI: 10.2–14.1), about threefold higher than the national prevalence of 4.04% (95%CI: 3.6–4.53) for persons aged 15–34 years in Kenya in 2012^30^. Coupled with the high prevalence of 14.9% (KAIS 2007)^31^ and 15.1% (KAIS 2012)^30^ reported for Nyanza, these statistics collectively emphasise the large HIV burden in this region, hence the need for well designed prevention strategies. As commonly observed in other parts of sub-Saharan Africa, HIV was more prevalent in females in this study than males^32^,^33^, potentially due to disproportionate social and biological factors influencing vulnerability as previously reported^34^, and possibly an imbalance in health seeking behaviours.

According to Kenya’s census projections from 2000 to 2020, the total population of persons aged 15–34 years in Kisumu in 2007 was 233,570^35^. Assuming that the 2007 HIV incidence rate among persons aged 15–34 years was 1.46% in Kisumu based on the MAA, approximately 3,400 persons in Kisumu aged 15–34 years had recent HIV infection. This is approximately 3% of the estimated number of recent HIV cases that occurred in Kenya among persons aged 15–49 years which ranged from 120,000 to 140,000 in 2007^36^. While this may not be an exact estimate due to design differences, it illustrates the case of ongoing HIV transmission in this area despite HIV prevention efforts. While HIV risk patterns appear similar between established and recent infections, symptomatic and diagnosed STI infections could be among key factors driving new HIV transmission in this younger population. This is consistent with HIV risk factor analysis reported previously^34^. Two recently published studies also identified history of STI as a risk factor for recent HIV infections in both rural western Kenya and the country as a whole^16^,^25^. Prompt diagnosis and treatment of STIs, accompanied with risk reduction counselling remain vital to the success of HIV prevention initiatives in this setting. Additional efforts are needed to share risk knowledge with younger adults who are sexually active and promote early HIV testing.

The overall distribution of HIV-1 subtypes in our study was synonymous to patterns earlier reported for this region^24^,^25^. Although slight changes were observed between recent and established infections, where some subtypes were apparently lower (A and D) or not found (AC, AG and G), while AD and C apparently increased among recent infections, the variations were not statistically significant, implying a mature epidemic. Additionally, Kenya is bordered by five countries with variable distribution of HIV-1 subtypes. Subtypes C and AC dominate in Somalia and Ethiopia, C and D in Sudan, while A and D are the most common subtypes in Uganda. Tanzania has subtypes A, C, D, AC, AD and CRF_CD in varying proportions^37^. Owing to the ongoing regional integration among East African states, frequent transfer of different viral subtypes between states is highly likely, hence continuous monitoring of HIV strains remains an important consideration when carrying out biological investigations in this region.

Although only polymorphic drug-resistance mutations, which have a low effect on HIV therapy, were observed in this study, such polymorphisms could lead to rapid treatment failure and development of drug resistant HIV-1 variants following initiation of therapy^38^. For instance, V82I polymorphism in subtype G contributes to emergence of I82M/T/S resistance after protease inhibitor based treatment failure^39^. The pattern of resistance mutations in this study could be a reflection of lack of prior ARV exposure. Nevertheless, with the increase in ARV use and consequent primary drug resistance mutations in Kenya as well as southern and eastern Africa regions, the importance of frequent drug resistance surveillance cannot be overstated^40^,^41^.

Finally, the small sample size of recent infections, and the convenience sampling method employed to screen participants for KICoS1, may have affected the statistical power of various variables. We also lacked professional panels to generate local incidence assays window periods. Although we utilised longitudinal KICoS1 incidence to validate the derived cross-sectional incidences, the possibility of misclassification by the MAA cannot be completely ruled out. These factors may reduce the representativeness of our findings.

## Conclusion

In summary, our study presents an MAA that estimated cross-sectional HIV incidence with perfect concordance to longitudinal incidence, with a mean recency of infection below one year. This offers important insights on the performance of MAAs in local African epidemics. This MAA allowed us to demonstrate the possibility of comprehensive evaluations covering key groups in the HIV epidemic, i.e. the HIV negative, recent and established infections. This study showed that current/past STI infections could be possible independent factors for new HIV infection in this population. We observed limited viral resistance mutations, four pure HIV-1 subtypes (A, C, D, and G) plus a number of recombinant viruses, and existence of transmission clusters, consistent with previous molecular surveys in this region. Application of our strategy in larger cross-sectional studies will enable a more in-depth assessment with definite outcomes that will support progressive approaches for tackling the spread of HIV.

## Methodology

### Study population

As previously described^27^, KICoS was an observational prospective cohort study that sought to longitudinally determine HIV incidence among sexually active 16–34 year olds in Kisumu, western Kenya. A total of 1106 individuals were screened for eligibility for recruitment from 2007 to 2009. Through convenience sampling, the study enrolled male or female residents of Kisumu aged 16–34 years, sexually active in the past three months, not pregnant, and HIV uninfected^27^. Demographic and behavioural information was collected at screening via Audio Computer Assisted Self Interview (ACASI), followed by medical examination and testing for common sexually transmitted infections (STIs). This was termed KICoS1, followed by later design modifications to target at-risk individuals (KICoS2 and 3)^42^. Procedures in this study were conducted in accordance with, and under the oversight of the approving ethical bodies. In the current study, we focus on the baseline data and samples collected from persons screened for participation in KICoS1.

### Sample collection

Whole blood samples were collected in vacutainer tubes with EDTA (Becton Dickinson, San Jose, CA, USA). Plasma was separated and stored frozen until use.

### Viral load measurements

HIV-1 plasma RNA VL was quantified using CobasAmpliprep/CobasTaqman HIV-1 test v.2.0 according to manufacturer’s instructions (Roche Diagnostic System Branchburg, NL, USA).

### Cross-sectional HIV incidence assays

HIV positive samples were tested by three incidence assays: BED-Capture enzyme immunoassay (BED-CEIA) (Calypte, USA; Sedia BioSciences, USA), Limiting antigen (LAg) avidity (Sedia BioSciences, USA), and Biorad avidity assay, modified from the Genetic Systems 1/2 + O ELISA (Bio-Rad Laboratories, Redmond, WA). The BED-CEIA and LAg avidity were performed according to manufacturer’s instructions as previously described^5^,^20^,^43^. Normalised optical density (OD-n) <0.8 and <1.5 represented recent infections on BED-CEIA and LAg avidity respectively, while values above the cut-offs were considered established. Same was done for avidity index (AI) <30% for Biorad avidity assay.

### HIV genetic sequencing

Protease (1–99 amino acids) and part of reverse transcriptase (1–250 amino acids) regions of HIV-1 were sequenced by a broadly sensitive in-house assay as previously described^44^. Briefly, HIV-1 RNA was extracted using QiaAmp Viral RNA mini kit following manufacturer’s instruction (QiagenInc, Chatsworth, CA). Using primers spanning the target *pol* region, RT-PCR and nested PCR were conducted sequentially followed by Big Dye Terminator sequencing and resolution using an ABI 3100 Genetic Analyser (Applied Biosystems, Foster City, CA, USA). The sequences were assembled with Sequencher v.3.1 (Genecodes, Ann Arbor, MI) and quality checks done using sequence quality assessment tool (SQUAT).

To assess genetic diversity, sequences were analysed by REGA HIV-1 subtyping tool v.3.0, and further compared with NCBI-BLAST and MEGA v.7.0. Sequences showing ambiguous subtyping were selected for recombination analysis using SimPlot software v3.5.5 in a 400 base pair (bp) sliding window with 20 bp increments.

We also investigated potential existence of transmission clusters by evaluating evolutionary relatedness between the sequences in MEGA v.7. We used pair-wise Tamura Nei 93 (TN93) model, assuming gamma distribution (shape parameter = 0.3305). Potential transmission clusters were defined as ≥2 sequences with ≤1.5% genetic distance and high bootstrap values (>95%) from 1000 re-samplings^45^,^46^. The trees were rooted using subtype K reference sequence (Los Alamos Database accession number AJ249239_CM_K).

HIV viral resistance mutations were assessed by the algorithm in the Stanford University HIV Drug Resistance Database and categorised according to the International AIDS Society–USA Drug Resistance Mutations Group December 2010 updates^47^.

### Statistical methods

From the incidence assay results, we evaluated the performance of each assay singly and in various MAAs with and without VL (cut-off of >1000 copies/mL)^48^. Samples with missing values by any of the four parameters (three incidence assays or VL) were excluded from this analysis. We derived mean duration of recency (w) and 95% confidence intervals (95% CI) for the three assays from a previous publication^49^, and estimated percent incidence by individual assays and MAAs as previously described^50^, assuming missing at random to adjust for the samples missing incidence test data. We transformed the five parameters’ data into binomial values according to their respective cut-offs and generated heat maps using PermutMatrix-1.9.3 to evaluate the classification of samples by the five parameters. We considered published incidence of 1.4%, derived from the longitudinal phase of KICoS1^42^, as a guide to select suitable MAA for subsequent analyses. Kappa coefficient and Pearson’s phi coefficient (ϕ), with their respective 95% CIs and p-values, were calculated to measure agreement between incidence assays. We further sought to characterise misclassification by the three incidence assays based on the optimised MAA.

To assess factors potentially associated with the MAA-identified recent infections, we fitted two models using logistic regression for both bivariate and multivariate analyses. One model assessed recent infection versus HIV negatives and another model assessed recent infections versus established infections. All variables with bivariate p-value ≤ 0.2 or set a priori were included in the final multivariable models. In the multivariable models, covariates were added one by one to assess their individual effect on the outcome, while controlling for other covariates as potential confounders. We used likelihood ratio statistical test to select the best models. Chi-square and t-test were used to compare proportions and means respectively, considering statistical significance as p-value ≤ 0.05. Statistical analyses were performed using STATA version 13.0 (STATA Corporation, College Station, Texas, USA).

### Ethical Approval

Ethical approval was obtained from the Ethical Review Committee of the Kenya Medical Research Institute (KEMRI) as well as the Institutional Review Board of the U.S. Centers for Disease Control and Prevention (CDC), Atlanta, GA, USA. Written informed consent (with assent obtained in addition to parental consent for minors) was obtained from participants.

## Additional Information

**Accession codes:** KX306376-KX306475.

**How to cite this article**: Otecko, N. *et al*. Viral and Host Characteristics of Recent and Established HIV-1 Infections in Kisumu based on a Multiassay Approach. *Sci. Rep*. **6**, 37964; doi: 10.1038/srep37964 (2016).

**Publisher's note:** Springer Nature remains neutral with regard to jurisdictional claims in published maps and institutional affiliations.

## Supplementary Material

Supplementary Information

## Figures and Tables

**Table 1:** General characteristics of HIV infected and uninfected study participants, Kisumu Incidence Cohort Study (KICoS): 2007–2009.

Characteristic	Total, N = 1106	HIV positive, n = 133	HIV negative, n = 973
Gender			
Male	534 (48.3)	35 (26.3)	499 (51.3)
Female	572 (51.7)	98 (73.7)	474 (48.7)
Age			
16–17	260 (23.5)	10 (7.5)	250 (25.7)
18–24	635 (57.4)	71 (53.4)	564 (58.0)
25–29	149 (13.5)	31 (23.3)	118 (12.1)
30–34	62 (5.6)	21 (15.8)	41 (4.2)
Ever attended school			
Yes	625/671 (93.1)	102/116 (87.9)	523/555 (94.2)
Highest level of education			
Primary	386/1053 (36.7)	60/118 (50.8)	326/935 (34.9)
Secondary	381/1053 (36.2)	38/118 (32.2)	343/935 (36.7)
Technical	65/1053 (6.2)	5/118 (4.2)	60/935 (6.4)
College	201/1053 (19.1)	15/118 (12.7)	186/935 (19.9)
University	20/1053 (1.9)	0	20/935 (2.1)
Marital status			
Single/never married	747/1099 (68.0)	57/131 (43.5)	690/968 (71.3)
Single/stable partner	86/1099 (7.8)	10/131 (7.6)	76/968 (7.9)
Married	219/1099 (19.9)	46/131 (35.1)	173/968 (17.9)
Separated/divorce	32/1099 (2.9)	9/131 (6.9)	23/968 (2.4)
Widowed	15/1099 (1.4)	9/131 (6.9)	6/968 (0.6)
Sex for gifts			
Yes	203/1024 (19.8)	19/122 (15.6)	184/902 (20.4)
Sex for money			
Yes	168/235 (71.5)	21/30 (70.0)	147/205 (71.7)
Any partners last 3 months			
Yes	137/197 (69.5)	17/28 (60.7)	120/169 (71.0)
Ever treated for STI last 3 months			
Yes	141/1096 (12.7)	33/132 (25.0)	108/964 (11.2)
Syphilis test result			
Positive	17 (1.5)	7 (5.3)	10 (1.0)
Negative	1089 (98.5)	126 (94.7)	963 (99.0)
HSV-2			
Positive	272 (24.6)	88 (66.2)	184 (18.9)
Negative	752 (68.0)	29 (21.8)	723 (74.3)
Indeterminate	82 (7.4)	16 (12.0)	66 (6.8)
Condom use			
Yes	435/967 (45.0)	43/110 (39.1)	392/857 (45.7)

Note: Denominators indicated for variables with missing values.

**Table 2:** Performance evaluation of different single incidence assays and multiassay algorithms (MAAs), Kisumu Incidence Cohort Study (KICoS): 2007–2009.

Assay/MAA	Recent, n (%)	w (95%CI)	Incidence-% (95% CI)
BED, n = 125			
BED only	19 (15.2)	300 (270–329)	2.53 (1.35–3.71)
BED + VL	16 (12.8)	300 (270–329)	2.13 (1.05–3.20)
BED + LAg + VL*	11 (8.8)	300 (270–329)	1.46 (0.58–2.35)
LAg, n = 128			
LAg only	17 (13.2)	184 (161–208)	3.60 (1.81–5.39)
LAg + VL	16 (12.5)	184 (161–208)	3.39 (1.65–5.12)
Biorad, n = 125			
Biorad only	25 (20.0)	293 (263–323)	3.40 (2.00–4.81)
Biorad + VL	23 (18.4)	293 (263–323)	3.13 (1.79–4.48)

Note: Number of persons in the survey = 1106, persons HIV negative = 973, persons HIV positive = 133. BED, BED-CEIA OD-n < 0.8; LAg, LAg avidity OD-n < 1.5; Biorad, Biorad avidity AI < 30%; VL, viral load > 1000 copies/mL; w, mean duration of recency in days; CI, confidence intervals. ^*^selected MAA.

**Table 3:** HIV viral load characteristics of samples classified as established by the MAA (N = 114) categorised by three single incidence assays, Kisumu Incidence Cohort Study (KICoS): 2007–2009.

	Recent		Established	
	n (%)	Viral load	n (%)	Viral load
Biorad < 30	15 (13.2)	81838.27	99 (86.8)	161325.55
BED < 0.8	8 (7.0)	40014.38	106 (93.0)	159232.91
LAg < 1.5	5 (4.4)	98989.40	109 (95.6)	153246.39

Note: There were no differences in HIV viral load between recent and established infections (t-test).

**Table 4:** Factors potentially associated with testing recent on MAA as compared to HIV negative persons, Kisumu Incidence Cohort Study (KICoS): 2007–2009.

Characteristic	Bivariate		Multivariate	
	OR^*^ (95% CI)	p-value	OR (95% CI)	p-value
Gender				
Male	1		1	
Female	1.84 (0.54–6.33)	0.332	1.42 (0.35–5.77)	0.625
Age				
24–34	1		1	
16–23	0.35 (0.10–1.14)	0.082	0.43 (0.11–1.64)	0.218
Sex for gifts				
No	1		1	
Yes	0.39 (0.05–3.06)	0.371	0.68 (0.08–5.75)	0.723
Ever treated for STI				
No	1		1	
Yes	6.60 (1.98–22.01)	0.002	3.94 (1.03–15.07)	0.045
Syphilis test result				
Negative	1		1	
Positive	0.046 (0.01–0.24)	0.001	10.15 (1.51–68.22)	0.017
HSV–2				
Negative	1		1	
Positive	4.91 (1.31–18.47)	0.019	2.64 (0.60–11.55)	0.199
Indeterminate	5.47 (0.98–30.47)	0.052	4.11 (0.68–25.00)	0.125

^¥^Odds ratios with 95% confidence intervals (CI).

**Table 5:** Characteristics of individuals with HIV resistance mutations, Kisumu Incidence Cohort Study (KICoS): 2007–2009.

Individual	Resistance mutation		HIV-1 subtype	Duration of infection	Gender
	PR	RT			
1	V82I/V	—	C	Recent	Female
2	V82I	V108I/V	AD	Recent	Male
3	V82I	—	G	Established	Female
4	—	K101Q	AD	Established	Female
5	—	K101Q	D	Established	Female

Abbreviations: PR = *protease* gene, RT = *reverse transcriptase* gene.
