# Supplementary material for: Viral and Host Characteristics of Recent and Established HIV-1 Infections in Kisumu based on a Multiassay Approach
Source: Sci Rep. 2016 Nov 29;6:37964. doi: 10.1038/srep37964 (PMC5126579; doi:10.1038/srep37964)
Supplement: Supplementary Information [file srep37964-s1.pdf]

Title: Viral and Host Characteristics of Recent and Established HIV-1 Infections in Kisumu based on a Multiassay Approach

Authors: Newton Otecko[1], Seth Inzaule[1], Collins Odhiambo[1], George Otieno[1], Valarie Opollo[1], Alex Morwabe[1], Kennedy Were[1], Kenneth Ndiege[1], Fredrick Otieno[1], Andrea A. Kim[2], Clement Zeh[2]*

Institutional Affiliations:

[1]Kenya Medical Research Institute/U.S. CDC Research and Public Health Collaboration (KEMRI/CDC) and KEMRI Center for Global Health Research, Kisumu, Kenya

[2]U.S. Centers for Disease Control and Prevention (CDC-Kenya, Division of HIV/AIDS Prevention) and the KEMRI/CDC Research and Public Health Collaboration, Kisumu,

***Corresponding Author**

Clement Zeh, PhD, MPH,

CDC – Ethiopia, U.S. Embassy

P.O. Box 1014 - Entoto Road

Addis Ababa, Ethiopia

Email: cbz2@.cdc.gov

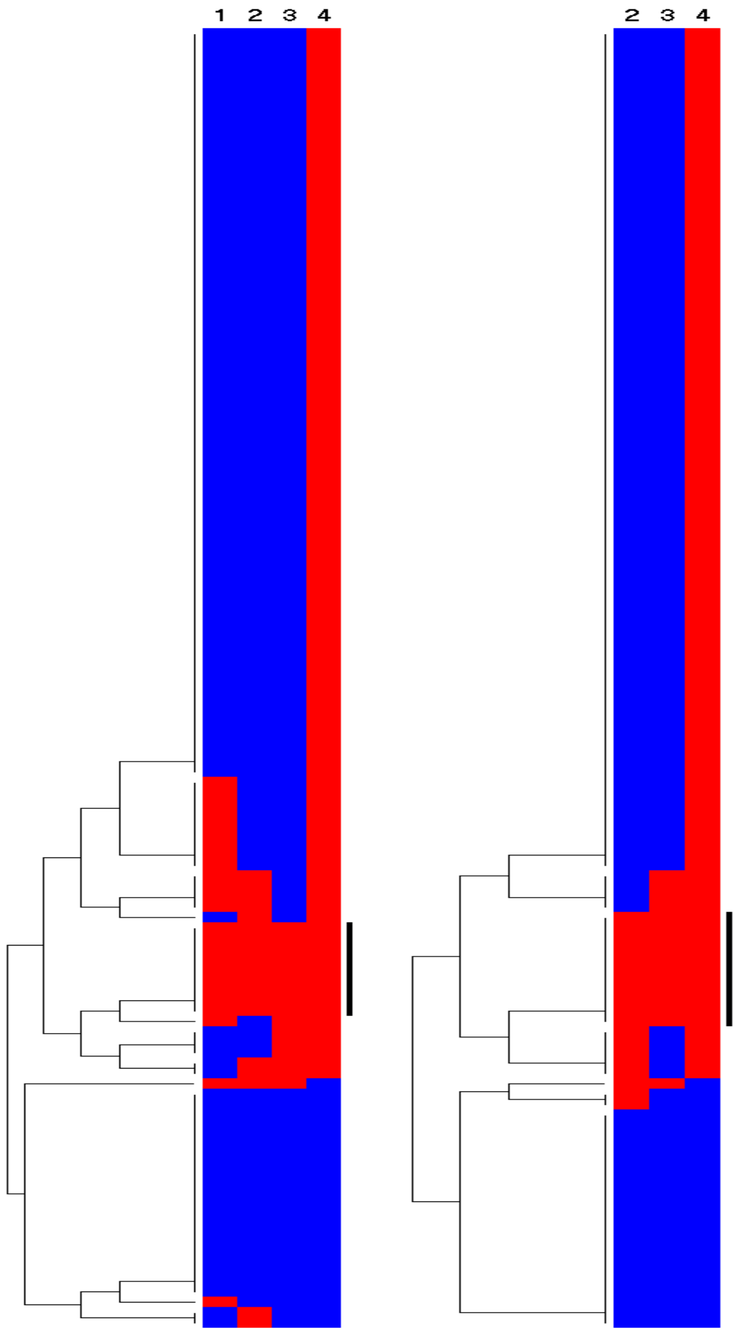

Supplementary Figure S1: Heat maps plotted for four (left) and three (right) assays used in testing for recent infections among HIV positive samples (N=125). Column numbers, 1: Biorad (AI<30%), 2: BED_CEIA (OD-n<0.8), 3: LAg avidity (OD-n<1.5), 4: viral load copies/mL (>1000). Red colour indicates positive (within cut-off) and Blue

colour negative (outside cut-off) for each parameter. Black bars on the right margins of the maps represent clusters classified as recent HIV infections.

Supplementary Table S1: Cross-tabulation of recent and established infections based on BED-CEIA and Limiting antigen (LAg) avidity assays, Kisumu Incidence Cohort Study (KICoS): 2007-2009

		LAg avidity assay		Total
		Recent	Established	
BED-CEIA	Recent	12	7	19
	Established	4	102	106
Total		16	109	125

Note: BED-CEIA versus LAg avidity: Kappa score = 0.635 (P = 0.001, 95% CI: 0.429-0.841), Pearson's phi coefficient (ϕ) = 0.638 (P = 0.001).

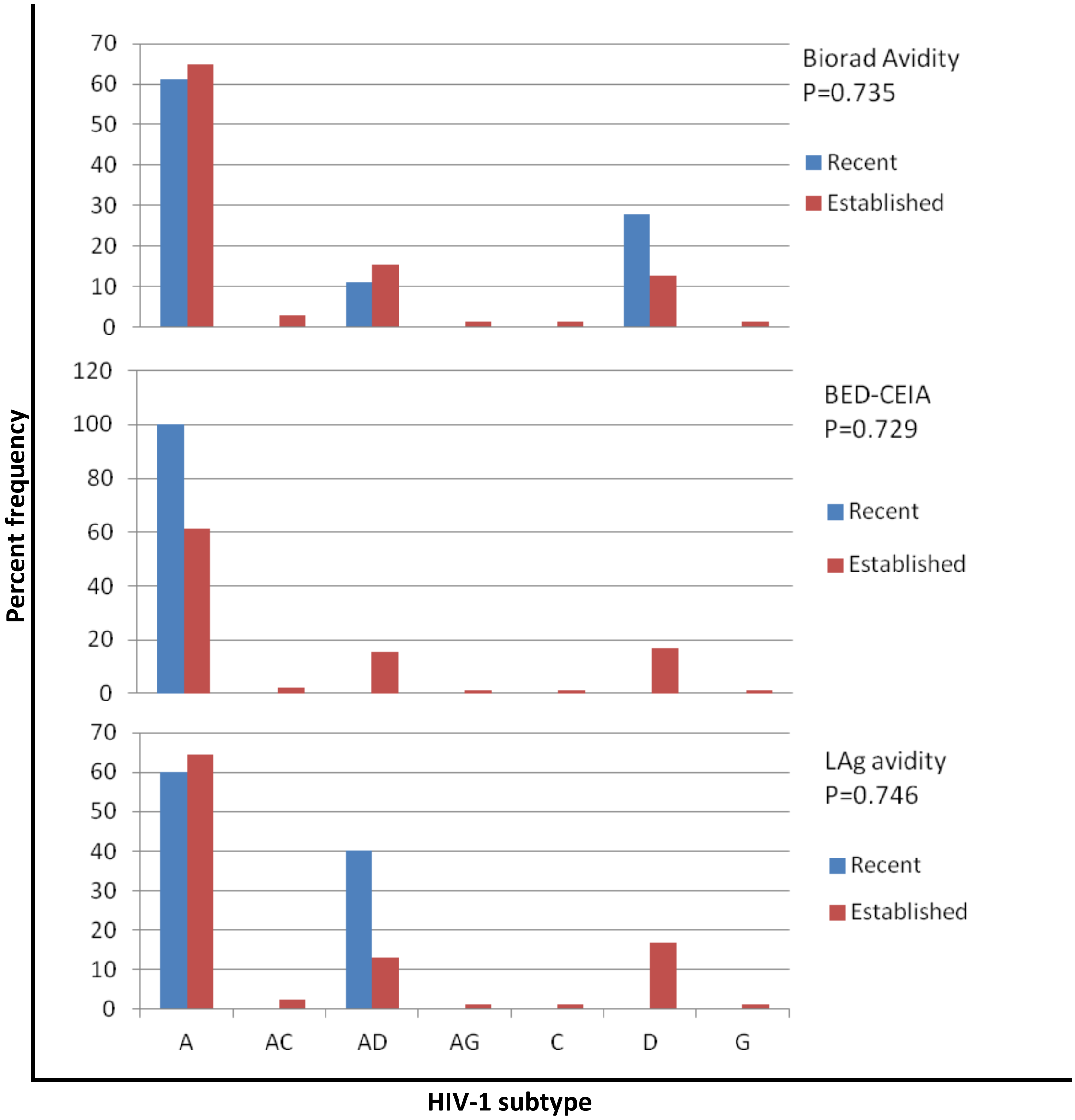

Supplementary Figure S2: HIV-1 subtypes of 89 of the 114 individuals classified as established having genotyping results. Bar graphs show percent subtype frequencies (Chi-square p-values indicated) among samples that would be termed recent and established infections as per Biorad avidity (top), BED-CEIA (middle) and LAg avidity (bottom) incidence assays.

Supplementary Table S2: General characteristics of HIV recent infections, Kisumu Incidence Cohort Study (KICoS): 2007-2009

Participant	HIV subtype	Viral load	Gender	Age	marital	Highest education
1	C	4004	female	27	single	College
2	AD	88660	male	20	single	College
3	A	512600	female	22	single	College
4	C	686400	male	27	single	nr*
5	D	376200	female	22	married	Primary
6	A	194700	male	24	separated/divorced	nr
7	A	86240	female	28	married	Secondary
8	A	17930	female	20	single	Secondary
9	AD	9482	female	21	single	Primary
10	A	278300	male	30	single	Primary
11	A	187000	female	16	single	Secondary

*no response. viral load in copies/mL.

Supplementary Table S3: Factors potentially associated with recent HIV infections as compared to persons with established infections, Kisumu Incidence Cohort Study (KICoS): 2007-2009

Characteristic	Bivariate		Multivariate	
	OR[¥] (95% CI)	p-value	OR (95% CI)	p-value
Gender				
Male	1		1	
Female	0.62 (0.17-2.29)	0.478	3.49 (0.38-31.95)	0.268
Age				
24-34	1		1	
16-23	0.97 (0.28-3.37)	0.964	0.45 (0.07-2.79)	0.389
Ever attended school				
No	1		1	
Yes	0.40 (0.07-2.24)	0.300	0.82 (0.07-10.20)	0.879
Sex for gifts				
No	1		1	
Yes	0.55 (0.07-4.60)	0.581	1.06 (0.09-12.11)	0.962
Ever treated for STI				
No	1		1	
Yes	3.09 (0.87 – 10.90)		9.91 (1.55-63.46)	0.015
Syphilis test result				
Negative	1		1	
Positive	4.84 (0.82-28.59)	0.081	7.67 (0.78-75.82)	0.081

HSV-2				
Negative	1		1	
Positive	0.35 (0.86-1.41)	0.139	0.27 (0.03-2.53)	0.252
Indeterminate	0.85 (0.14-5.28)	0.858	0.43 (0.02-8.71)	0.585

¥Odds ratios with 95% confidence intervals (CI). ***** Separated/divorce/widowed.

Supplementary Table S4: Characteristics of individuals belonging to HIV transmission clusters, LAg avidity, Kisumu Incidence Cohort Study (KICoS): 2007-2009

Variable	Dyad 1		Dyad 2	
	Individual 1	Individual 2	Individual 1	Individual 2
Viral load (copies/mL)	5269	56320	305800	1276000
HIV subtype	A	A	AD	AD
Gender	Female	Male	Female	Male
Age	26	32	22	28
Education	Primary	Primary	Primary	Secondary
Marital status	Married	Married	Single	Single
Partners ≤3 months (n)	3	1	1	1
Past STIs treatment	No	No	Yes	Yes
Syphilis	Negative	Positive	Negative	Negative
HSV-2	Positive	Positive	Positive	Positive
Condom use	Yes	No	Yes	No
